# Evaluation of Anticonvulsant, Sedative, Anxiolytic, and Phytochemical Profile of the Methanol Extract from the Aerial Parts of *Swertia corymbosa* (Griseb.) Wight *ex* C.B. Clarke

**DOI:** 10.1155/2014/542385

**Published:** 2014-04-27

**Authors:** G. Mahendran, G. Thamotharan, S. Sengottuvelu, V. Narmatha Bai

**Affiliations:** ^1^Department of Botany, School of Life Sciences, Bharathiar University, Coimbatore, Tamilnadu 641046, India; ^2^Department of Pharmacology, Nandha College of Pharmacy and Research Institute, Erode 638052, India

## Abstract

The objective of the present study was to evaluate the anxiolytic, antidepressant, and anticonvulsant activity of the methanolic extract of *Swertia corymbosa* (SCMeOH). After acute toxicity test, oral treatment with SCMeOH at doses of 125, 250, and 500 mg/kg behavioral models of open field, elevated-plus-maze, actophotometer, rotarod, pentylenetetrazole, isoniazid, and maximal electroshock induced seizure models were utilized. In open field test, SCMeOH (125, 250, and 500 mg/kg) (*P* < 0.01, *P* < 0.001) increased the number of rearings. However, the number of central motor and ambulation (*P* < 0.01, *P* < 0.001) were reduced. Likewise, the number of entries and the time spent in open arm were increased while the number of locomotion was decreased (*P* < 0.001) in elevated-plus-maze and actophotometer test, respectively. SCMeOH (125–500 mg/kg) protected the mice against the pentylenetetrazole and isoniazid induced convulsions; it causes significant (*P* < 0.01 and *P* < 0.001) dose dependent increase in latency of convulsion. Treatment with SCMeOH reduced the duration of the tonic hind limb extension induced by electroshock. Two major compounds such as gentiopicroside and swertianin were analyzed by HPLC system.

## 1. Introduction

Mental disorders affect 450 million people worldwide, with 121 million suffering from depression, and fifty million people are suffering from epilepsy worldwide. Eighty percent of epilepsy patients are living in the developing countries, where three-fourths of the patients are not receiving adequate treatment [1]. According to National Commission on Macroeconomics and Health report, nearly 65–70 million people in India have some kind of mental illness [2].

Anxiety is a highly prevalent psychological and physiological state characterized by psychomotor tension, sympathetic hyperactivity, and apprehension and vigilance syndromes and affecting one-eighth of the total population of the world and became a very important area of research interest in psychopharmacology [3]. Synthetic anxiolytic drugs such as benzodiazepines (BDZ), diazepam (DZP), and buspirone (BUSP) are considered as the main category of compounds prescribed for treatment of anxiety disorders. Unfortunately, they have several side effects such as tolerance, amnesia, weakness, loss of sexual drive, gastrointestinal effects and changes in body weight, sedation, muscle relaxation, and physical dependence, which lead patients to seek alternative therapies [4]. In an attempt to resolve these issues, interest has increased in alternative plant-related drugs. Several studies have shown that several ethnomedicinal/traditional medicinal plants have been documented for the treatment of central nervous system (CNS); these ethnomedicinal plants could serve as sources of effective medication that may be more readily accessible and inexpensive and thus would be helpful in improving the present status [5, 6].

The genus* Swertia* (family: Gentianaceae) is used in the Indian and Chinese traditional system of medicine since prehistoric times. It comprises about 170 species worldwide, of which 40 species are present in India. Xanthones, secoiridoids, and triterpenoids are the main constituents of the* Swertia* species. The extracts of a number of species have long been used in folk medicine for the treatment of hepatitis, cholecystitis, pneumonia, dysentery, scabies, spasm, pain, and neurasthenia.* Swertia corymbosa *(SC) is a pharmacologically unexplored, highly bitter species and is frequently mixed with* S. chirayita* as a substitute and/or adulterant [7]. It is an ethnomedicinal plant used for nervous disorders and sedation [8, 9]. SC has been reported for analgesic, anti-inflammatory, antioxidant, antiproliferative, and antidiabetic activities [10–13]. SC has been extensively studied for its phytochemical constituents, especially for the xanthones, iridoid, secoiridoid glycosides, and terpenoids such as oleanolic acid, ursolic acid, lupeol, and *β*-amyrin have been isolated from different parts of SC [13–15]. Early researchers found the neuropharmacological activity of some xanthones, such as gentiacaulein and gentiakochianin [16, 17] which also have been found in* Swertia corymbosa*. However, there is still no direct scientific report for its neuropharmacological effect. So the present investigation was designed to evaluate the anxiolytic, sedative, and anticonvulsant activity of the* Swertia corymbosa *using animal models, with a view to establish the pharmacological basis for its neurological disorder uses in folk medicine.

## 2. Materials and Methods

### 2.1. Plant Collection and Authentication

The aerial parts of* Swertia corymbosa* (Griseb.) Wight* ex* C.B. Clarke were collected from the state of Tamilnadu, India, in October 2010. The plant was identified and authenticated by Dr. R. Ramachandran, a plant taxonomist at Bharathiar University, Tamilnadu, India, where a voucher specimen with the number BU-006144 was deposited.

### 2.2. Preparation of Extracts

The plant material was dried in the dark at room temperature and powered. Five hundred grams of the powdered aerial parts of the* Swertia corymbosa* (SCMeOH) was extracted with 1.5 l of methanol (100%) in a soxhlet apparatus for 72 h. The methanol extract was dried by removal of the solvent under vacuum. The yield of SCMeOH was found to be 13.65% w/w.

### 2.3. Drugs and Chemicals

Isoniazid (S. D. Fine Chemicals); diazepam (Calmpose Inj. Ranbaxy, India); pentylenetetrazole (Sigma, USA); methanol extra pure (Rankem).

### 2.4. Animals

Young male albino Swiss mice (18–25 g) and Wistar albino rats (150–200 g) were obtained from the Animal Centre, Department of Pharmacology, Nandha College of Pharmacy and Research Institute, and were housed in plastic cages at room temperature with a 12 : 12 h light-dark cycle. They were fed with balanced rodent pellet diet and water* ad libitum*. The animals were acclimatized for at least 1 week before being used for experiments. All the studies conducted were approved by the Institutional Animal Ethics Committee of Nandha College of Pharmacy and Research Institute, Department of Pharmacology, and the approval number was 688/02/C/CPCSEA.

### 2.5. Acute Toxicity Test

Acute toxicity of SCMeOH was evaluated according to the method described by Organization of Economic Cooperation and Development Guideline 423 [18]. The animals were kept fasting for overnight providing only water. The SCMeOH was administered orally at a dose of 5 mg/kg (suspended in 0.5% carboxymethyl cellulose (CMC)) initially to separate groups of mice and mortality was observed for 3 days. If mortality was observed in 4/6 or 6/6 animals, then the dose administered was considered as toxic dose. However, if the mortality was observed in only 1 mouse out of 6 animals, then the same dose was repeated with higher doses such as 50, 300, 500, 1000, and 2,000 mg/kg b.w. orally. The control mice were given 10 mL/kg of water. The mice were observed for behavioural changes and mortality within 24 h. After this, daily observations for toxicity and mortality were made up to the 14th day.

### 2.6. Anticonvulsant Activity

#### 2.6.1. Pentylenetetrazole Induced Convulsions

Pentylenetetrazole (PTZ) induced convulsions test was performed to evaluate anticonvulsant property of drugs [19]. Thirty male mice were divided into five groups, each group comprised six mice. Different groups were treated with distilled water (10 mL/kg), diazepam (5 mg/kg), and SCMeOH at doses of 125, 250, and 500 mg/kg, BW. Thirty minutes later, convulsions were induced by the intraperitoneal administration of 60 mg/kg BW of PTZ. Following the administration of PTZ, mice were placed in separate transparent Plexiglas cages (25 × 15 × 10 cm) and were observed for the occurrence of seizures over a 30 min time period. Latency of convulsions (the time prior to the onset of tonic convulsions), duration of tonic convulsions, and mortality protection (percentage of deaths in 24 h) were recorded [20].

#### 2.6.2. Maximal Electroshock (MES) Induced Convulsions

The animals were divided into five groups, each group comprised six rats. Different groups were treated with distilled water (10 mL/kg), diazepam (5 mg/kg), and ScMeOH at doses of 125, 250, and 500 mg/kg, BW. Thirty minutes later, convulsions were induced in all the groups of animals using electroconvulsometer. A 60 Hz alternating current of 150 mA for 2 s was delivered through the ear electrodes [21]. The animal was observed for the occurrence of tonic hind limb extension.

#### 2.6.3. Isoniazid (INH) Induced Convulsions

Thirty male mice were divided into five groups, each group comprised six mice. Different groups were treated with distilled water (10 mL/kg), diazepam (5 mg/kg), and SCMeOH at doses of 125, 250, and 500 mg/kg, BW. Thirty minutes later, convulsions were induced by the intraperitoneal administration of 250 mg/kg BW of INH. During the next 30 min time period, latency of the first clonic convulsion, duration, and mortality protection (percentage of deaths in 24 h) were recorded [22].

### 2.7. Assessment of Sedative Activity

#### 2.7.1. Spontaneous Motor Activity

The locomotor activity was performed as per Adnaik et al. [23] through actophotometer. The movement (i.e., the number of light-beam crossings) of the animal interrupts a beam of light falling on a photocell, at which a count was recorded and displayed digitally. Each rat was placed individually in the actophotometer for 10 min and the basal activity score was obtained. Subsequently, the animals were divided into five groups, each consisting of six animals. Different groups were treated with distilled water (10 mL/kg), diazepam (2 mg/kg), and SCMeOH at doses of 125, 250, and 500 mg/kg, BW. After 60 min the rat was placed again in the actophotometer for observing the activity for 10 min [23].

### 2.8. Rotarod Performance

The equipment of Rotarod was used to evaluate motor coordination produced by drugs in animals [24]. The mice were trained before the experiment to acquire the capacity to remain for 300 s on a diameter rod, rotating at 20 rpm. Two or three trials were sufficient for the animals to learn this task. Thirty mice were divided into five groups; each group comprised six rats. Different groups were treated with distilled water (10 mL/kg), diazepam (5 mg/kg), and SCMeOH at doses of 125, 250, and 500 mg/kg, BW. Then, the animals were placed in the four paws on the rotating bar, which is 2.5 cm in diameter and 25 cm high from the floor. The animals were observed for a period of five minutes. The difference between the fall-off time of the mice before and after treatment was considered as an index of muscle relaxation [24, 25].

### 2.9. Anxiolytic Test

#### 2.9.1. Elevated Plus Maze (EPM) Test

The EPM test is the most frequently employed model for the assessment of the anxiolytic activity of novel substances [26]. The elevated plus maze apparatus consisted of two perpendicular open arms (50 × 10 cm) and two perpendicular enclosed arms (50 × 10 × 40 cm). The entire maze was constructed of wood and elevated 50 cm above floor. The maze was placed inside a light (25 lx) and sound attenuated room.

The animals were divided into five groups, each group comprised six rats. Different groups were treated with distilled water (10 mL/kg), diazepam (5 mg/kg), and SCMeOH at doses of 125, 250, and 500 mg/kg, BW. Thirty minutes later, the rat was placed in the center platform of the maze facing the enclosed arm and was observed for 10 min. The parameters assessed were the time spent in open and enclosed arms and numbers of open and enclosed arms entries. All tests were taped by using a video camera and every precaution was taken to ensure that no external stimuli could evoke anxiety in the mice. After each test, the maze was carefully cleaned up with a wet tissue paper (70% ethanol solution) to eliminate the interference of the olfactory cues on the next rat [27].

### 2.10. Open Field Test

The study was conducted according to method previously described by Brown et al. [28] with some modifications. The apparatus was made up of plywood measuring 72 cm × 72 cm × 36 cm. One of the walls was made of transparent Perspex glass to ensure that the mouse under investigation is visible to the observer. The floor, made of cardboard, was divided into 16 equal squares (18 cm × 18 cm) with blue marker and a central square drawn with black marker. The cardboard was covered with a transparent Plexiglas. The animals were divided into five groups; each group comprised six rats. Different groups were treated with distilled water (10 mL/kg), diazepam (5 mg/kg), and SCMeOH at doses of 125, 250, and 500 mg/kg, BW. Thirty minutes later, each mouse was placed individually at the corner of the arena and its behavior monitored for 5 min. The number of rearings, ambulation, and central locomotion by each mouse was recorded. The apparatus was wiped between observations with 70% ethyl alcohol and allowed to dry to remove any olfactory cue.

### 2.11. HPLC Analysis

We performed an HPLC analysis of SCMeOH for detection and quantification of its major constituents. A Beckman 125 HPLC instrument (Beckman, USA) equipped with a Hamilton auto sampler and a 168 photodiode array detector (DAD) was used and the detection wavelength was set at 254 nm. Separation was carried out using an ODS-C18 column (150 mm × 4.6 mm, 5 *μ*m). The injection volume was 10 *μ*L and elution was performed at a flow rate of 0.8 mL/min with the following solvent ratios for the mobile phase where solvent A is 1% acetic acid and solvent B is acetonitrile. The gradient elution was as follows: 0–8 min, 2–15% (B), 8–20 min, 15% (B), and 20–50 min, 15–100% (B). We used gentiopicroside internal reference and swertianin was prepared from our previous work and identified by comparing the ^1^H-NMR, ^13^C-NMR, and 2D NMR (H-H COSY, C-H COSY, and HMBC) data with the literature [10]. Quantification of the main compounds was achieved using calibration curves that were separately constructed with pure standards [29].

### 2.12. Data Analysis

Results of the experiments and observations were expressed as mean ± standard deviation (SD). The significance of differences between groups was determined using one-way analysis of variance (ANOVA) followed by at least one of the following post hoc tests: Dunnett's multiple comparison tests *P* < 0.05 where level of significance was considered for each test. The data is presented as mean ± S.D.

## 3. Results

### 3.1. Acute Toxicity Test

The methanol extract of* Swertia corymbosa* (SCMeOH) did not produce any mortality orally up to 2000 mg/kg, but the mice manifested signs of sedation like quiescence and reduced locomotion at high doses when observed for 5 h after administration. There were no visible signs of delayed toxicity and mortality observed when the animals were monitored for a further 14 days.

### 3.2. Anticonvulsant Activity

#### 3.2.1. PTZ Induced Convulsion

Pentylenetetrazole produced tonic seizures in the entire animals used. A dose of 125 mg/kg of ScMeOH protected 33.33% of the animals against seizures and did not affect the onset (latency) of seizures to any significant extent. SCMeOH at the dose of 250 and 500 mg/kg protected 50.0% and 100% of the mice against seizures and significantly (*P* < 0.01 and *P* < 0.001) increased the latency of the seizures (Table 1).

#### 3.2.2. INH Induced Convulsion

Isoniazid (250 mg/kg, i.p.) elicited clonic convulsions in all the animals used. The normal control group produced convulsion and showed latency of 89.20 ± 4.24 s. The methanol extract of* S. corymbosa* (125–500 mg/kg) significantly (*P* < 0.01 and *P* < 0.001) delayed the duration of ionized induced seizures from 56.77 ± 0.19 s in control to 41.30 ± 0.20 s, 34.62 ± 1.55 s, and 20.99 ± 2.24 s, respectively and shows dose dependent increase in the anticonvulsant activity. Similarly, Diazepam 5 mg/kg pretreatment significantly (*P* < 0.001) increased the latency of isoniazid induced seizures from 56.77 ± 0.19 s to 527.11 ± 6.11 s with 100% protection (Table 2).

#### 3.2.3. MES Model

Maximal electroshock produced hind limb tonic extension (HLTE) in all the animals. The vehicle treated rats showed tonic hind limb extension for duration of 12.88 ± 0.35 s. Administration of SCMeOH (125–500 mg/kg) showed a dose dependent increase in the delay of the onset time of seizures induced by maximal electroshock induced convulsion and also decreased duration of tonic hind limb extension (Table 3).

### 3.3. Measurement of Locomotor Activity

In actophotometer reading, locomotor activity of the plant extract treated was dose dependently reduced (Table 4).

### 3.4. Rotarod Performance

Table 5 shows the effects of SCMeOH from* S. corymbosa* in the Rotarod test, a method used for evaluating motor coordination and presence of any muscle griping effect. It revealed that there was significantly increased grip force and fall time after administration of SCMeOH (125, 250, and 500 mg/kg) when compared to control. All the SCMeOH treated animals retained on the rotating rod for more than 276.35 ± 7.58 s at 500 mg/kg as shown in Table 5, indicate SCMeOH to be devoid of neurotoxicity.

### 3.5. Elevated Plus Maze (EPM)

**Figure 1:**
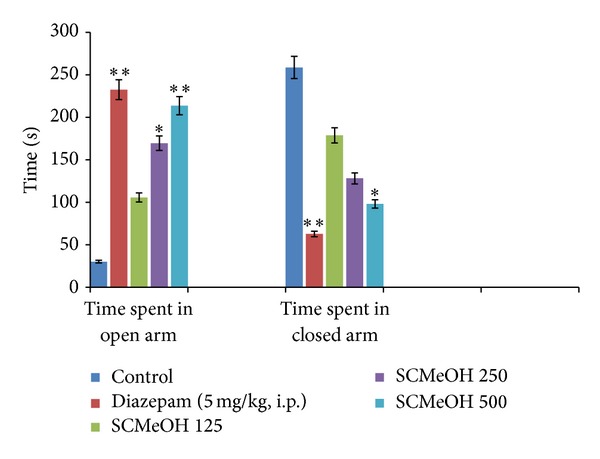
Effect of oral administration of* Swertia corymbosa* methanolic extract (SCMeOH) on the time spent in open arms (TOA) and time spent in closed arm (EOA) by rat exposed to EPM. *P < 0.01 and **P < 0.001 indicate significant difference from control.

**Figure 2:**
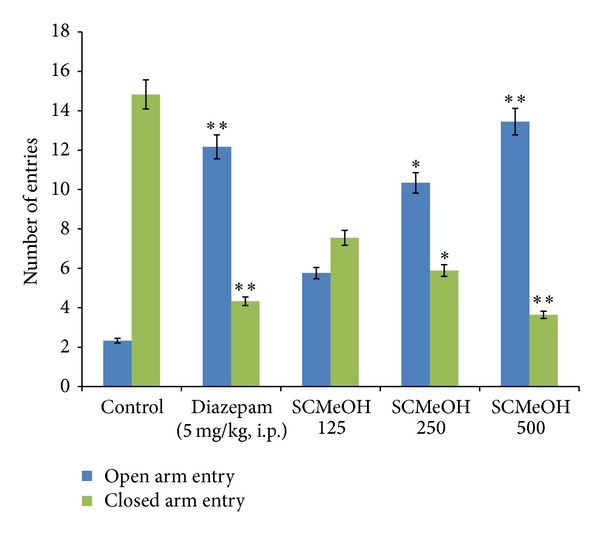
Effect of oral administration of* Swertia corymbosa* methanolic extract (SCMeOH) on the number of entries in open and closed arms by rats exposed to EPM. *P < 0.01 and **P < 0.001 indicate significant difference from control.

**Figure 3:**
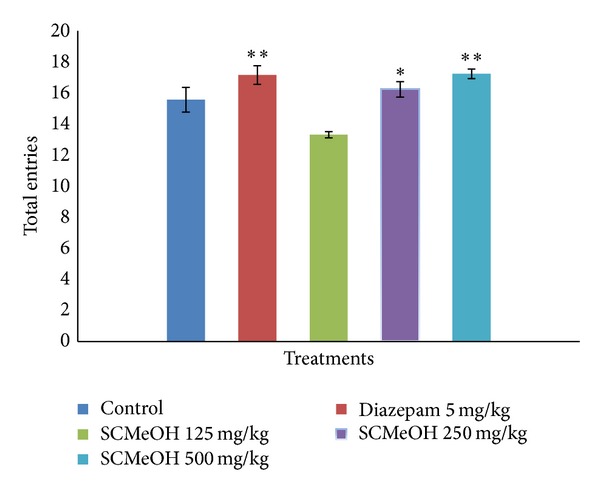
Effect of oral administration of* Swertia corymbosa* methanolic extract (SCMeOH) on the total of entries in open and closed arms by rats exposed to EPM. *P < 0.01 and **P < 0.001 indicate significant difference from control.

Administration of SCMeOH (125–500 mg/kg) to the rats causes the significant (*P* < 0.01 and *P* < 0.001) increase in the frequency of the open arm entries (Figure 1). Significant and dose dependent increase in the duration of time spent in the open arm was observed in SCMeOH (125–500 mg/kg) treated rats. Extract at doses of 125–500 mg/kg produce a low number of entries in the closed arm, while control (10 mL/kg) had the highest closed arm entry value of 14.83 ± 0.33 (Figure 2). The effects of SCMeOH (125–500 mg/kg) and diazepam resulted in significant increases in the total number of entries into the two arms (*P* < 0.01 and *P* < 0.001) (Figure 3).

### 3.6. Open Field Test (OFT)

**Figure 4:**
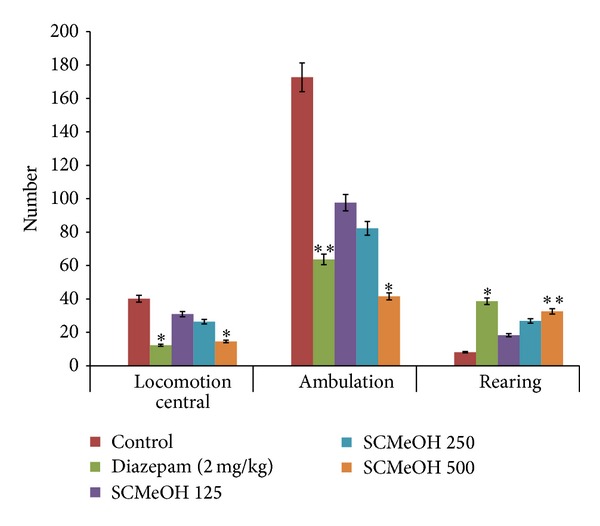
Effect of oral administration of* Swertia corymbosa* methanolic extract (SCMeOH) on the central locomotion ambulation and rearings of mice exposed in open field test. *P < 0.01 and **P < 0.001 indicate significant difference from control.

Administration of SCMeOH (125–500 mg/kg BW) to the mice causes the statistically significant reduced number of central motor and ambulation. However, diazepam (2 mg/kg) and SCMeOH increased the number of rearings (Figure 4).

### 3.7. Phytochemical Analysis of SCMeOH Using HPLC Analysis

**Figure 5:**
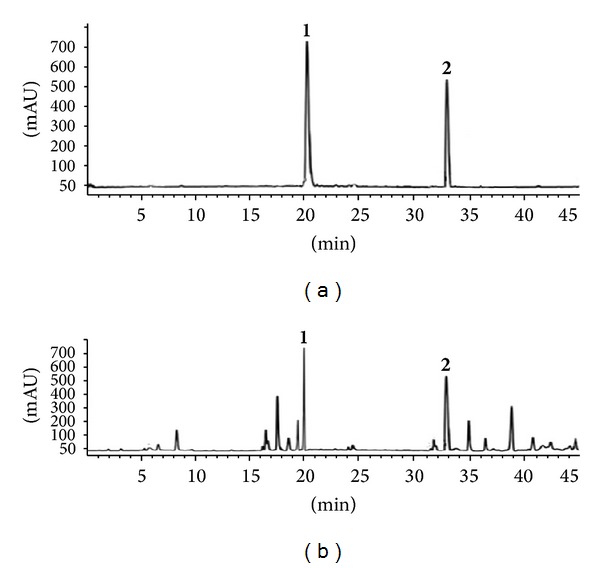
(a) HPLC—chromatograms of gentiopicroside and swertianin. (b) HPLC—chromatograms of methanol extract from* Swertia corymbosa* (SCMeOH).

**Figure 6:**
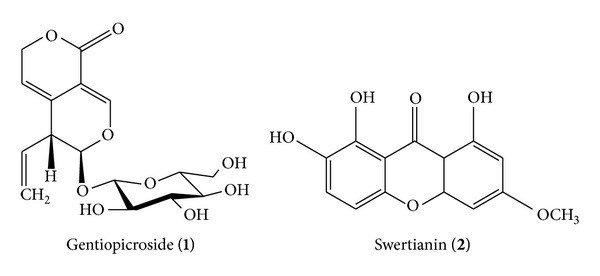
Structures of gentiopicroside and swertianin.

We analyzed SCMeOH by HPLC for detection and quantification of major constituents of active extract.  Figure 5 shows the HPLC profile of SCMeOH recorded at 254 nm, in which 10 peaks were detected. The main compounds of SCMeOH, peak 1 (*t*_*R*_ = 20.0 min) and peak 2 (*t*_*R*_ = 32.8 min), (spectral data and characterization of swertianin provided as in the supplementary file in the Supplementary Material available online at http://dx.doi.org/10.1155/2014/542385) were identified and quantified as gentiopicroside (40.726 mg/g) and swertianin (29.598 mg/g), respectively (Figure 6), since the spectroscopic data were found to be in good agreement with values reported in the literature [10, 13, 15]. Quantification established with calibration curves of both gentiopicroside and swertianin had a good linear relationship. The liner ranges of gentiopicroside and swertianin were 0.0653–8.0542 mg/mL (*Y* = 511,072*x* + 13,124.0, *n* = 5, *R* = 0.9995) and 0.228–1.14 mg/mL (*Y* = 135,827*x* − 1, 719.1, *n* = 5, *R* = 0.9995), respectively.

## 4. Discussions

Medicinal plants have served as sources of readily accessible, inexpensive, and effective medication since the earliest times known to man. Several ethnomedicinal plants have been found to possess neurobehavioral profile and serve as alternative to modern medicine. Biological evaluation and scientific validation of the ethnomedicinal plants are the need of the hour [30, 31]. The present study was proposed to assess CNS depressant, anxiolytic, and anticonvulsant effects of methanolic extract of aerial parts of an ethnomedicinal plant,* Swertia corymbosa*.

The results of the present laboratory animal study indicate that* S. corymbosa* SCMeOH possesses anticonvulsant activity. This observation is in agreement with the findings of Srivastava et al. [17] who recently reported the neurological studies of* Swertia chirayita* (Roxb. ex. Flem) Karsh, another species and member of the Gentianaceae family, in mice and rats. The present study demonstrated the anticonvulsant effects of the methanol aerial parts extract of* S. corymbosa* in both chemically and electrically induced seizures in mice and rats. The extract exhibited dose dependent protection in the MES, PTZ, and INH tests. Further, like diazepam, the extract provides 100% protection at 500 mg/kg in the INH and PTZ models. Nevertheless, in unprotected animals, the extract significantly increased seizure latency and reduced seizure duration compared with the control group in all three models at all tested doses. The effect of most of antiepileptic agents is to enhance the response to GABA by facilitating the opening of GABA-activated chloride channels. GABA_A_ receptors were involved in epilepsy and their direct activation would have an antiepileptic effect. It is well documented that PTZ/NIH induced convulsions are produced due to alteration of GABA level in the brain. Therefore, SCMeOH might possibly be producing an antiepileptic action by increasing the level of GABA, an inhibitory transmitter in the central nervous systems. This is in accord with the pharmacological effects of benzodiazepine and highlights the relevance of the putative antiepileptic effects of SCMeOH [23].

In the present study, sedative effect of* S. corymbosa* (SCMeOH) is evaluated by using a locomotor activity test. Locomotor activity is considered as an index of alertness and a decrease in it is indicative of sedation. The antiepileptic effects of drugs such as benzodiazepines are accompanied by decreased locomotor activity and sedation. The extract inhibited locomotor activity in this experiment. SCMeOH inhibited locomotor activity to a lesser extent than diazepam and thus has a better profile for an antiepileptic effect. The neuropharmacological activities of SCMeOH such as sedative and anticonvulsant effect are similar to the central depressant properties of extract of plants such as* Rubus brasiliensis, Valeriana adscendens, Trema cannabina*,* Heteropterys brachiate,* and* Moringa oleifera *[1, 32–35].

In the present work, a clear anxiolytic-like activity of SCMeOH has been observed. The elevated plus maze has been frequently used to detect and evaluate anxiolytic/anxiogenic-like properties of drugs. The frequency and time spent in open arms are the major indexes of anxiety in plus maze model, given the fact that rodents are extremely aversive to an open area [36]. Diazepam increases the number of entries into the open arms and increases the time spent in the open arms, demonstrating the characteristic anxiolytic effect of BDZ compounds. Results of this study indicated that the extract has a selective anxiolytic effect with significantly modifying the locomotor activity like other herbal extracts where anxiolytic activity is accompanied by sedative and locomotor effects and these include* Xeromphis niclotica* [37],* Palisota hirsute* [38],* Balanites aegyptiaca *[39],* Viscum album* [40], and* Zizyphus nummularia *[6].

The anticonvulsant, anxiolytic, and sedative effects of benzodiazepines like diazepam are mostly attributed to enhance the action of gamma-aminobutyric acid (GABA_A_) [41]. Actually, benzodiazepines bind to the gamma subunit of the GABA_A_ receptor, due to which a structural modification of the receptor results in an increase in GABA_A_ receptor activity. Benzodiazepines do not substitute for GABA, which bind at the alpha subunit, but increase the frequency of channel opening events, which leads to an increase in chloride ion conductance and inhibition of the action potential [42, 43]. According to some researchers, the anxiolytic action of benzodiazepines may be due to the direct activation of glycine synapses in the brain [42, 44]. This may explain the mechanism of action of our tested extract as well, because it is clear from the results that the effect of the extract was similar to diazepam.

Previous phytochemicals reported in the literature, various xanthones, iridoids, secoiridoid glycosides, and triterpenoids, isolated from* Swertia* species would be the effective constituents. In the present study, we identified the presence of two major compounds: gentiopicroside and swertianin in SC methanol extract by HPLC analysis. Gentiopicroside is secoiridoids and swertianin is a xanthone. Previous studies have also proved that xanthones like alkanolamides, alkanolamines, and aminoalkanolic have shown anticonvulsant activity [45]. The anxiolytic and antidepressant effect of gentiacaulein and gentiakochianin has been previously reported [17] and therefore, we suggest that these anxiolytic effects may be due to the above constituents. Our research group is undertaking the project of the isolation of active constituents from* S. corymbosa* whole plant.

In conclusion, SCMeOH possesses anxiolytic, sedative and convulsant effects and these findings collaborate with the ethnomedicinal uses of this plant. The isolation of active chemicals from this plant might serve as lead compounds for the synthesis of drugs which could be used in the management of these nervous disorders.

## Supplementary Material

The phytochemicals present in most *Swertia* species have been found to be iridoids, xanthones, mangiferin and C-glucoflavones. Xanthones are the major class of compounds among the chemical constituents of this genus. Xanthones are yellow pigments characteristic of some higher plant families, including Gentianaceae and Guttiferae. The iridoids (mainly secoiridoid glucosides) appear to be present in all species investigated with a predominance of swertiamarin, sweroside and gentiopicroside. The distribution of iridoids has been shown to have considerable value as a systematic character since they occur almost exclusively in *Swertia* species.

## Figures and Tables

**Table 1:** Effect of methanol extract of *Swertia corymbosa* (SCMeOH) on pentylenetetrazole induced seizure.

Treatment (mg/kg)	Latency of tonic convulsion (sec)	Duration of tonic convulsion (s)	Mortality (% death)	% protection
Control	100.20 ± 3.34	446.10 ± 5.19	6/6 (100)	0.0
Diazepam (5 mg/kg, i.p.)	478.34 ± 6.07**	126.69 ± 1.93**	0/6 (0.0)	100
SCMeOH 125	141.43 ± 1.98	216.29 ± 1.23	4/6 (66.66)	33.33
SCMeOH 250	298.16 ± 4.45*	189.19 ± 1.72*	3/6 (50.00)	50.0
SCMeOH 500	416.42 ± 6.14**	137.11 ± 2.61**	0/6 (16.66)	100

Control (vehicle)—distilled water.

The data represent the mean ± SD (*n* = 6). *P < 0.01, **P < 0.001 significantly different compared to PTZ induced seizure control.

**Table 2:** Effect of methanol extract of *Swertia corymbosa* (SCMeOH) on INH induced seizure in mice.

Treatment (mg/kg)	Latency of tonic convulsion (s)	Duration of convulsion (s)	Mortality (% death)	% protection
Control	89.20 ± 4.24	56.77 ± 0.19	6/6 (100)	0.0
Diazepam (5 mg/kg, i.p.)	527.11 ± 6.11**	26.65 ± 0.93**	0/6 (0.0)	100
SCMeOH 125	301.82 ± 5.43	41.30 ± 0.20	2/6 (33.33)	66.6
SCMeOH 250	441.67 ± 2.10*	34.62 ± 1.55*	1/6 (16.66)	83.32
SCMeOH 500	511.12 ± 2.11**	20.99 ± 2.24**	0/6 (0.00)	100

Control (vehicle)—distilled water.

The data represent the mean ± SD (*n* = 6). *P < 0.01, **P < 0.001 significantly different compared to NIH induced seizure control.

**Table 3:** Effect of methanol extract of *Swertia corymbosa* (SCMeOH) on tonic seizures induced by maximal electroshock in rats.

Treatment (mg/kg)	Seizure onset time (s)	Duration of tonic hind limb extension (s)
Control	8.38 ± 1.88	12.88 ± 0.35
Diazepam (5 mg/kg, i.p.)	19.88 ± 1.35	2.63 ± 1.72**
SCMeOH 125	28.81 ± 1.10	8.28 ± 1.19
SCMeOH 250	32.43 ± 1.44*	7.44 ± 1.01*
SCMeOH 500	48.84 ± 1.25**	3.21 ± 1.25**

Control (vehicle)—distilled water.

The data represent the mean ± SD (*n* = 6). *P < 0.05, **P < 0.01 significantly different compared to maximal electroshock induced seizure control.

**Table 4:** Effect of methanol extract of *Swertia corymbosa* (SCMeOH) on locomotor activity (actophotometer) in rats.

Treatment (mg/kg)	Locomotor activity in 10 min		% change in activity
	Before	After 60 minutes	
Control	294.5 ± 16.69	281.23 ± 11.43	
Diazepam (2 mg/kg)	289.56 ± 6.23	145.96 ± 3.25	49.59**
SCMeOH 125	287.51 ± 9.17	180.12 ± 2.34	37.35
SCMeOH 250	299.50 ± 10.62	162.60 ± 2.49	45.70*
SCMeOH 500	298.44 ± 12.20	133.16 ± 3.19	55.38**

Control (vehicle)—distilled water.

The data represent the mean ± SD (*n* = 6). *P < 0.01, **P < 0.001 significantly different compared to normal control.

**Table 5:** Effect of methanol extract of *Swertia corymbosa* (SCMeOH) on motor (rotarod) coordination in mice.

	Experimental mean time (10 min) (s)		% of muscle griping
	Before	After 60 minutes	
Control	69.16 ± 2.18	67.00 ± 2.89	
Diazepam (2 mg/kg)	64.73 ± 5.82	13.30 ± 7.42	—
SCMeOH 125	70.23 ± 3.12	127.19 ± 4.32	40.78
SCMeOH 250	65.78 ± 2.89	199.56 ± 2.43	67.03*
SCMeOH 500	70.12 ± 1.22	276.35 ± 7.58	74.62**

Control (vehicle)—distilled water.

The data represent the mean ± SD (*n* = 6). *P < 0.01, **P < 0.001 significantly different compared to normal control and diazepam.
