# Supplementary material for: Evaluation of Anticonvulsant, Sedative, Anxiolytic, and Phytochemical Profile of the Methanol Extract from the Aerial Parts of Swertia corymbosa (Griseb.) Wight ex C.B. Clarke
Source: Biomed Res Int. 2014 Apr 27;2014:542385. doi: 10.1155/2014/542385 (PMC4022199; doi:10.1155/2014/542385)
Supplement: Supplementary file 1 — The phytochemicals present in most Swertia species have been found to be iridoids, xanthones, mangiferin and C-glucoflavones. Xanthones are the major class of compounds among the chemical constituents of this genus. Xanthones are yellow pigments characteristic of some higher plant families, including Gentianaceae and Guttiferae. The iridoids (mainly secoiridoid glucosides) appear to be present in all species investigated with a predominance of swertiamarin, sweroside and gentiopicroside. The distribution of iridoids has been shown to have considerable value as a systematic character since they occur almost exclusively in Swertia species. [file 542385.f1.pdf]

<u>Supplementary Information</u>

Evaluation of anticonvulsant, sedative, anxiolytic and phytochemical profile of the methanol extract from the aerial parts of *Swertia corymbosa* (Griseb.) Wight *ex* C.B. Clarke

G. Mahendran [a*], G. Thamotharan[b] S. Sengottuvelu[b], V. Narmatha Bai [a]

Supplementary figures

Swertianin-H-NMR

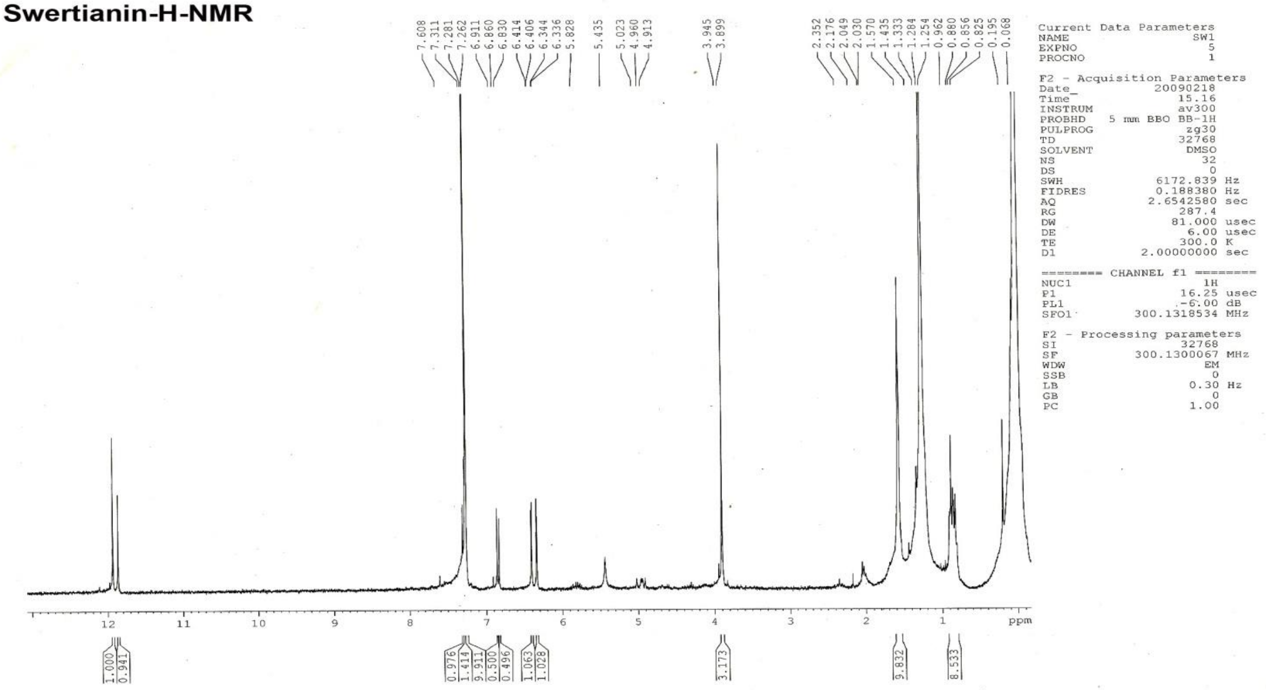

^{13}C-NMR spectra of swertianin

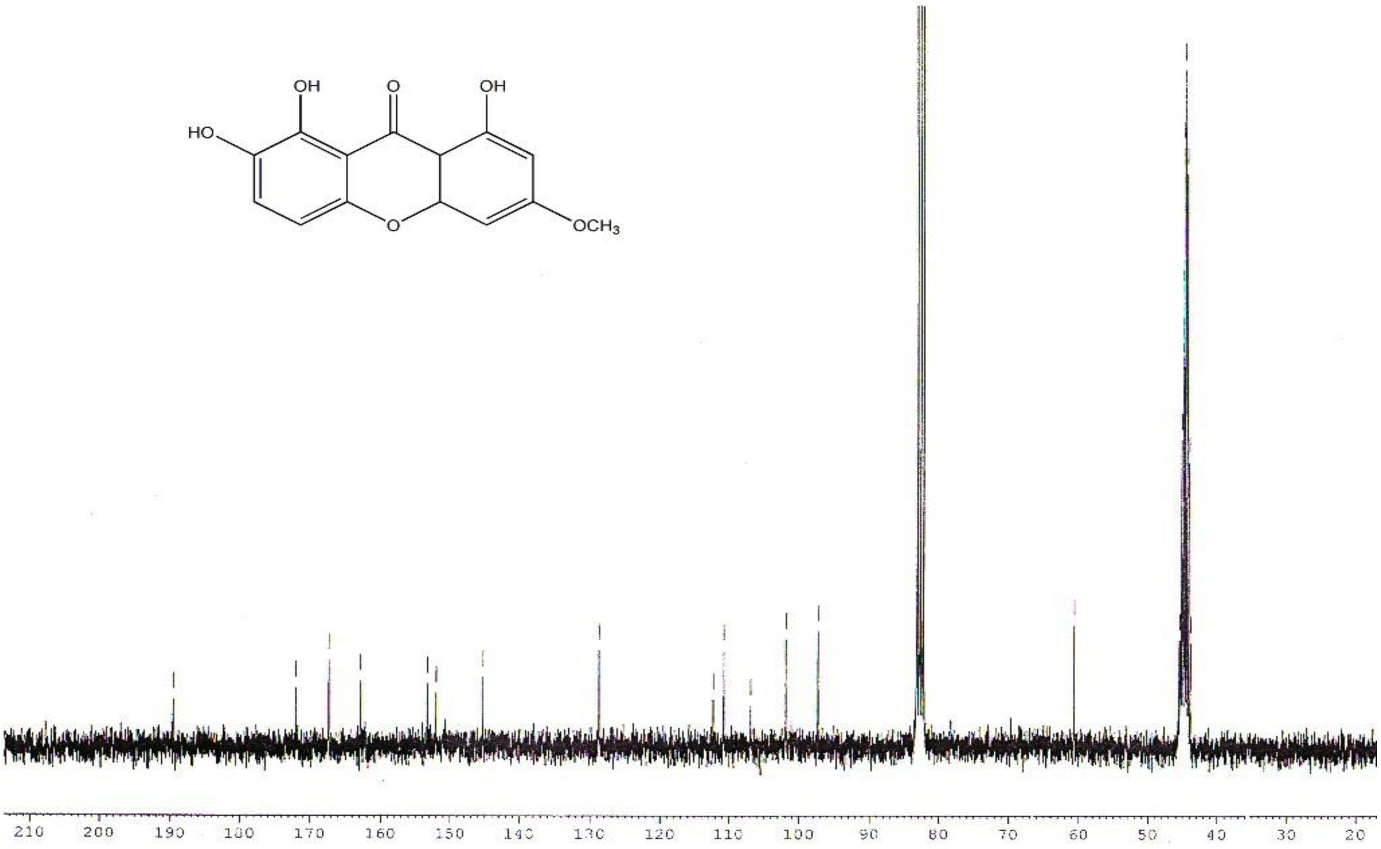

HMBC spectra of swertianin

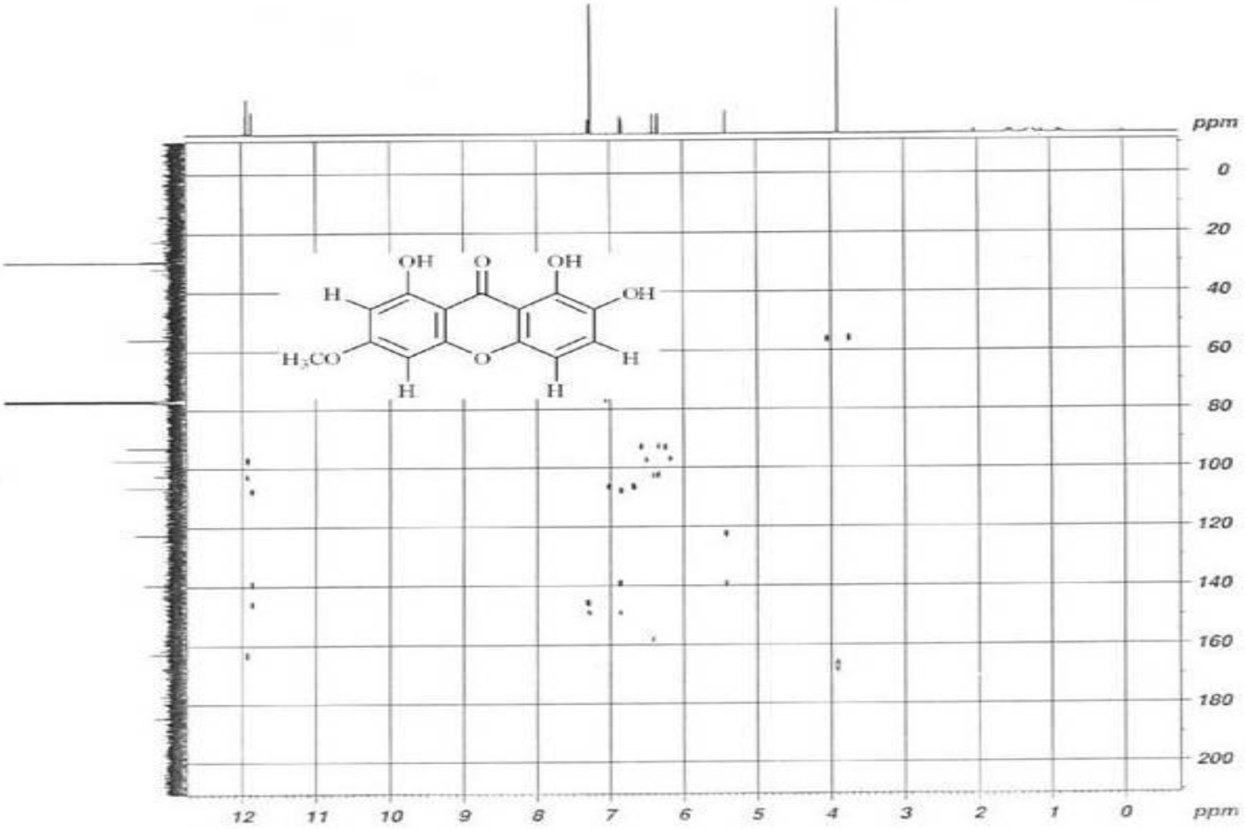

MS spectra of swertianin

National Centre for Mass Spectrometry
Indian Institute of Chemical Technology

Sample Information

Sample ID	: SW1	Name: G MAHENDRAN,RESEARCH SCHOLAR,Dept OF BOTANY,BHARATHIYAR UNIVERS
Data File	: E:\ISO\18774-1.QGD	
Analyzed by	: Admin	
Analyzed	: 3/16/2009 6:19:43 PM	
Tuning File	: C:\GCMSsolution\System\Tune1\Auto Tuning-EI(WithOUT Column)- 3-3-09.qgt	

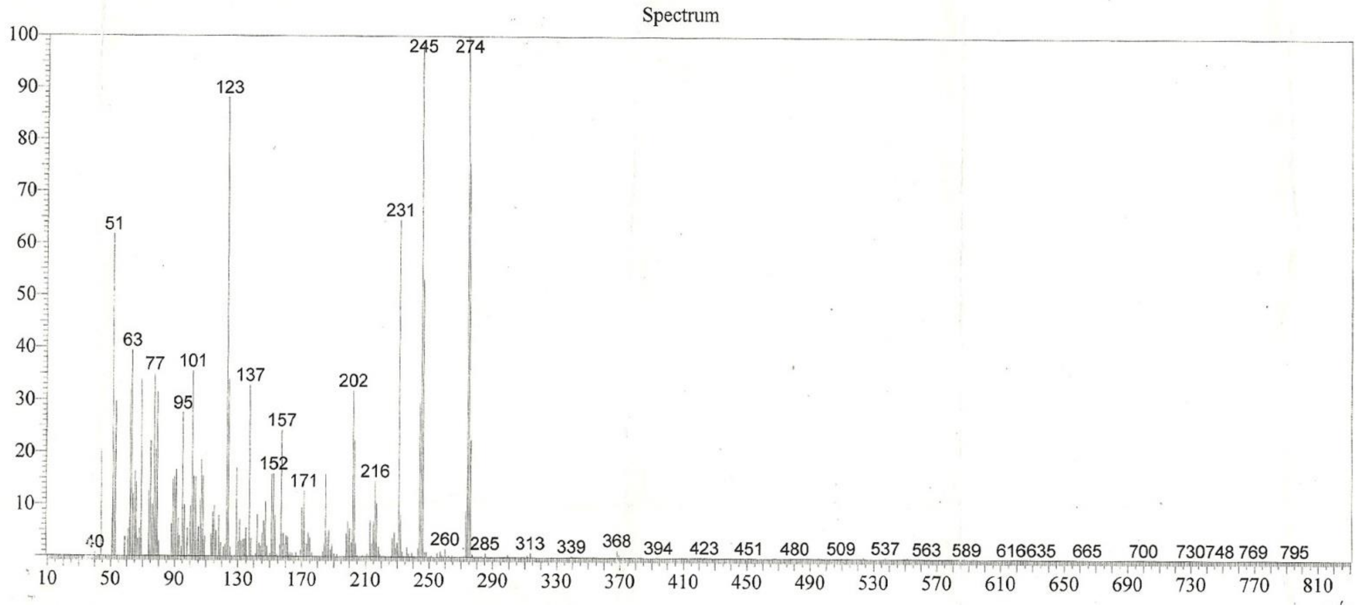

Compound 2	: Swertianin
Melting point	: 224–227 °C
IR (KBr) ν_{max} cm^{-1}	: 3388-3442 (OH groups), 1662 (C=O), 1282 ($O\text{-}CH_3$)
^1H NMR (300 MHz, $CDCl_3$) δ (ppm)	: 3.90 (s, 3H, $C_6\text{-}OCH_3$) 5.44 (b s, 3H, C_2-OH), 6.34 (d, 1H, J =2.40 Hz, H-5) 6.41 (d, 1H, J = 2.40 Hz, H-7) 6.85 (d, 1H, J =9.00 Hz, H-3), 7.30 (d, 1H, J =9.00 Hz, H-4), 11.87 (s, 1H C_8-OH) 11.94 (s, 1H C_1-OH).
^{13}C NMR (75 MHz, $CDCl_3$) δ (ppm)	: 60.69 ($C_6\text{-}OCH_3$), 97.33 (C_4), 101.84 (C_2), 106.89 (C_{9a}), 110.77 (C_5), 112.30 (C_{8a}), 128.75 (C_6), 145.23 (C_{10a}), 152.01 (C_{4a}), 153.28 (C_7), 162.79 (C_8), 167.27 (C_1), 171.92 (C_3), 189.51 (C=O).
HMBC	: H-3(C_1, C_{4a}), H-4(C_2, C_{4a}),H-5(C_6),H-7(C_5,C_8,C_{8a}), 1-OH(C_{9a}), 2-OH(C_3),6-OCH_3(C_6).
EIMS: *m/z*(%)	: 274 (M^+, 100 %) 260 (5 %), 246 (50 %), 245 (96 %), 231 (64 %), 216 (13 %), 202 (30 %) 185 (16 %), 171 (14 %), 157 (23 %), 152 (18 %), 137 (34 %), 123 (88 %), 101 (36 %), 98 (30 %), 77 (35 %), 63 (40 %), 51 (62 %). $C_{14}H_{10}O_6$ (274). Anal. Cal Found C: 61.46 %, H: 3.52 %.

1, 2, 8-Trihydroxy-6-methoxy xanthone (swertianin)

Bright yellow crystal, mp 224–227 °C, IR (cm^{-1}): 3388-3442 (OH groups), 1662 (C=O), 1282 (O-CH_3), EIMS m/z 274 $[M]^+$ (calculated for $C_{14}H_{10}O_6$). ^1H NMR ($CDCl_3$, 300MHz) 3.90 (s, 3H, C6-OCH_3) 5.44 (b s, 3H, C2-OH), 6.34 (d, 1H, J =2.40 Hz, H-5) 6.41 (d, 1H, J = 2.40 Hz, H-7) 6.85 (d, 1H, J =9.00 Hz, H-3), 7.30 (d, 1H, J =9.00 Hz, H-4), 11.87 (s, 1H C8-OH) 11.94 (s, 1H C1-OH). ^{13}C-NMR ($CDCl_3$, 75MHz) 60.69 (C6-OCH_3), 97.33 (C4), 101.84 (C2), 106.89 (C9a), 110.77 (C5), 112.30 (C8a), 128.75 (C6), 145.23 (C10a), 152.01 (C4a), 153.28 (C7), 162.79 (C8), 167.27 (C1), 171.92 (C3), 189.51 (C=O). HMBC: H-3(C1, C4a), H-4(C2, C4a),H-5(C6),H-7(C5,C8,C8a), 1-OH(C9a), 2-OH(C3),6-OCH_3(C6).
